# The relationship between explicit and implicit personality: Evidence from the Big Five and trait emotional intelligence

**DOI:** 10.1371/journal.pone.0287013

**Published:** 2023-10-09

**Authors:** Nasser N. Hasan, Konstantinos V. Petrides, Laura Hull

**Affiliations:** 1 Department of Clinical, Educational, and Health Psychology, University College London, London, United Kingdom; 2 Educational Psychology Department, College of Education, Kuwait University, Kuwait, Kuwait; 3 London Psychometric Laboratory, University College London, London, United Kingdom; 4 Centre for Academic Mental Health, Department of Population Health Sciences, Bristol Medical School, University of Bristol, Bristol, United Kingdom; Polytechnic Institute of Coimbra: Instituto Politecnico de Coimbra, PORTUGAL

## Abstract

The main aim of this study is to introduce an implicit personality assessment method (e.g., implicit association test) to Kuwait. We adapted an existing personality-related implicit association test (IAT; Big Five IAT), while also constructed the first trait EI IAT based on Petrides’ four-factor model. We investigated the psychometric properties of the implicit association test through assessing the reliability of scores and also their relationship with their corresponding explicit measures. The measures were administered to 1458 university students in Kuwait. The zero-order correlations showed that the explicit and implicit measurement approaches led to non-converging constructs in the case of both trait EI and the Big Five. Lastly, we believe that we were successfully able to introduce the concept of personality-related implicit association tests to the Kuwaiti sample. Subsequently, the IATs presented in our study will allow researchers to study a relatively new personality field, that is the implicit personality.

## Introduction

Historically, psychologists interested in studying the personality differences among individuals relied on self-report measures. These measures are direct and scientifically categorised under explicit measures. Explicit measures assess mental structures and processes accessible through introspection [1]. In other words, participants are asked directly to respond to items concerning their explicit needs, motives, values, and traits. For example, when you ask people directly how they feel about a product, you are using an explicit question.

In contrast, when you do not ask people directly about their feeling but assess their behaviour or how they perform a task (e.g., whether they come back to buy the same product again), you are using an indirect method. This indirect measurement method is referred to as implicit measure. Implicit measures are contrary to their explicit counterparts in which the earlier assess the mental structures and processes that are inaccessible through introspection [1]. Accordingly, one may define implicit measures as an indirect assessment tool that people may be unaware of or are unwilling to report [2]. Undoubtedly, response latencies are the most common implicit measure during the last decade.

In response to latency studies, researchers are interested in measuring the reaction times when participants perform a certain task. Thus, the participants are not asked about their feelings, instead, they focus on performing an objective task in which inferences are drawn from their timed performance.

Implicit association tests, known as IAT [3], are the most common response latency that relies on reaction times to assess different personality aspects such as attitudes and traits. Their main advantage over their explicit counterparts is that they are less susceptible to faking [4, 5]. It is obvious as people tend to distort their explicit feelings, attitudes, or traits to present themselves to others favourably. Accordingly, IAT is developed to make it hard for participants to control their responses (i.e., to fake their responses) and reveal things that people may not even know that they possess.

Petrides and Furnham [6] studied the EI construct and pointed out that different measurements lead to different constructs (e.g., trait EI vs. ability EI). We also argue that the implicit and explicit measures refer to two different personality-related constructs based on the measurement method: implicit personality and explicit personality. Although one method (i.e., implicit measure) overcomes the disadvantages of the other (i.e., explicit measure), we believe that they complement each other and neither one is superior. As suggested by James and LeBreton [1], researchers should study both aspects to develop a comprehensive understating of one’s personality and the consequential behaviours or constructs. More importantly, Lane and colleagues [7] presented a table comprising the correlation indices between an implicit measure and its corresponding explicit measure. Compared to what we argued, the correlation between the implicit and its corresponding explicit measure was weak in the majority of cases.

### Implicit association test

The IAT was introduced by Greenwald and colleagues [3] to assess implicit attitudes. Two years later, Greenwald and Farnham [8] introduced this measurement method to the personality field. The key concept behind the IAT is that inferences about one’s attitudes, feelings, or traits are based on reactions time (i.e., the time taken to perform the task). The logic behind relying on reaction times is based on the idea that people perform better (i.e., with speed and accuracy) when the task is aligned with their cognitive associations. In other words, when the task demands conflict with one’s automatic mental links, the test takers are slowed down and make more mistakes. In short, it is based on the association between the participant’s reaction time to categorise stimuli related to two pairs of concepts: *target* and *attribute*.

**Fig 1:**
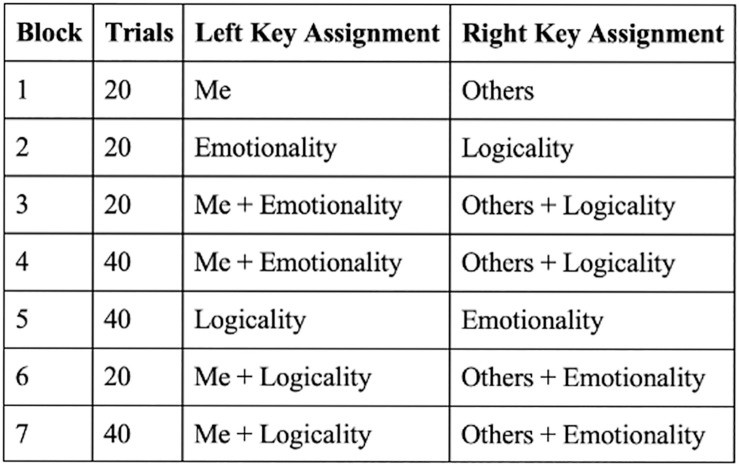
Schematic overview of the IAT.

The IAT test comprises five separate categorisation tasks, represented by seven blocks. In the first categorisation task (Block 1), the participant is asked to sort words relating to the concepts (e.g., Me and Others) into categories. In the second task (Block 2), participants are asked to perform the same sorting task, but this time with different concepts (e.g., Emotionality and Logicality). In the third task (Blocks 3 and 4), the categories are combined in a way that presents two concepts on the left of the screen, while the other two on the right (e.g., Me + Emotionality and Others + Logicality). In the fourth task (Block 5), the placement of the concepts presented in Block 2 switches (e.g., Logicality on the left and Emotionality on the right) and the participant is asked to perform the same sorting task. In the fifth task (Blocks 6 and 7), all concepts are combined again, but in a different combination than in Blocks 3 and 4 (e.g., Me + Logicality and Others + Emotionality). Noteworthy, in the subsequent three IAT subtests, the first categorisation task is eliminated as it will already have been presented in the first subtest (i.e., in the Me and Others categorisation task). An illustration of these blocks is presented in Fig 1 using one of the trait EI sub-IATs from our study, which was based on the guidelines by Lane et al. [7].

In concepts such as the Big Five and trait EI, several IATs, each called sub-IAT, are used to assess the underlying constructs. For instance, the Big Five IAT consists of five sub-IATs corresponding to the five constructs representing the Big Five [9]. Similarly, the trait EI IAT consists of four sub-IATs corresponding to the four factors of trait EI [10].

In both concepts, the first categorisation task was only presented in the first sub-IAT and eliminated from the following sub-IATs for two reasons. First, the participant has already been introduced to the concepts related to the first categorisation task (i.e., *Me + Others*) which is one of this IAT’s aims. Second, to avoid adding unnecessary trials that lead to longer tests, as adding these trials will result in including 140 more trials. Clearly, adding these trials will increase the likelihood of respondent fatigue [11], which can threaten the validity of our results.

### Building IAT blocks

As shown in Fig 1, the IAT comprises seven blocks. In Block 1, the participants are asked to rapidly classify stimulus (in our case, a word) into the concept *me* (by pressing the left assigned key “E” in English keyboard and “ث” in Arabic keyboard) and *others* (by pressing the right assigned key “I” in English keyboard and “هـ” in Arabic keyboard). The same task is repeated in Block 2 with two different concepts, *emotionality* and *logicality*. In Block 3, the previous two tasks are combined, and the participants are asked to perform the classification task with two concepts on each side; when the stimulus belongs to the concepts *me* or *emotionality*, the participant will have to press the left key. The same task is performed in Block 4 but with more trials. In Block 5, the task in Block 2 is reversed. The participants will press the left key if the stimulus belongs to the *logicality* concept and the right key for the *emotionality* concept. In the last two blocks, Block 6 and 7, the concepts are reversed from Blocks 3 and 4, and participants are asked to perform the same classification task.

Several considerations must be concerned when constructing an IAT. The first consideration is related to defining the construct because this will affect the choices of the categories in the next stage. Many categories have an obvious comparison category, such as *me* and *others* categories, in our study. However, in some cases, choosing the comparable category is not an obvious step. In such cases, it is advised to use a mutually exclusive category from the same domain. For example, Grumm and Collani [12] used *extraversion* and *introversion* categories in their study. Therefore, we followed the advice to use an approach to choose the appropriate categories in our study.

After choosing the appropriate categories, the IAT developer must ensure that the stimuli under each category are well-chosen. Lane et al. [7] stated *stimuli matter* when they discussed the contradictory attitudes toward America when the category America was presented by the names of certain presidents compared to flag images and other common sightseeing. Furthermore, they suggested avoiding negated stimuli because participants tend to take more time to process the negations and classify it properly, which indeed affects the response time. We believe that the stimuli must undergo pilot testing in which participants should be given the four categories and a shuffled list of all stimuli and asked to perform the classification task. By doing this, we can ensure whether the stimuli list under each category is appropriate.

**Fig 2:**
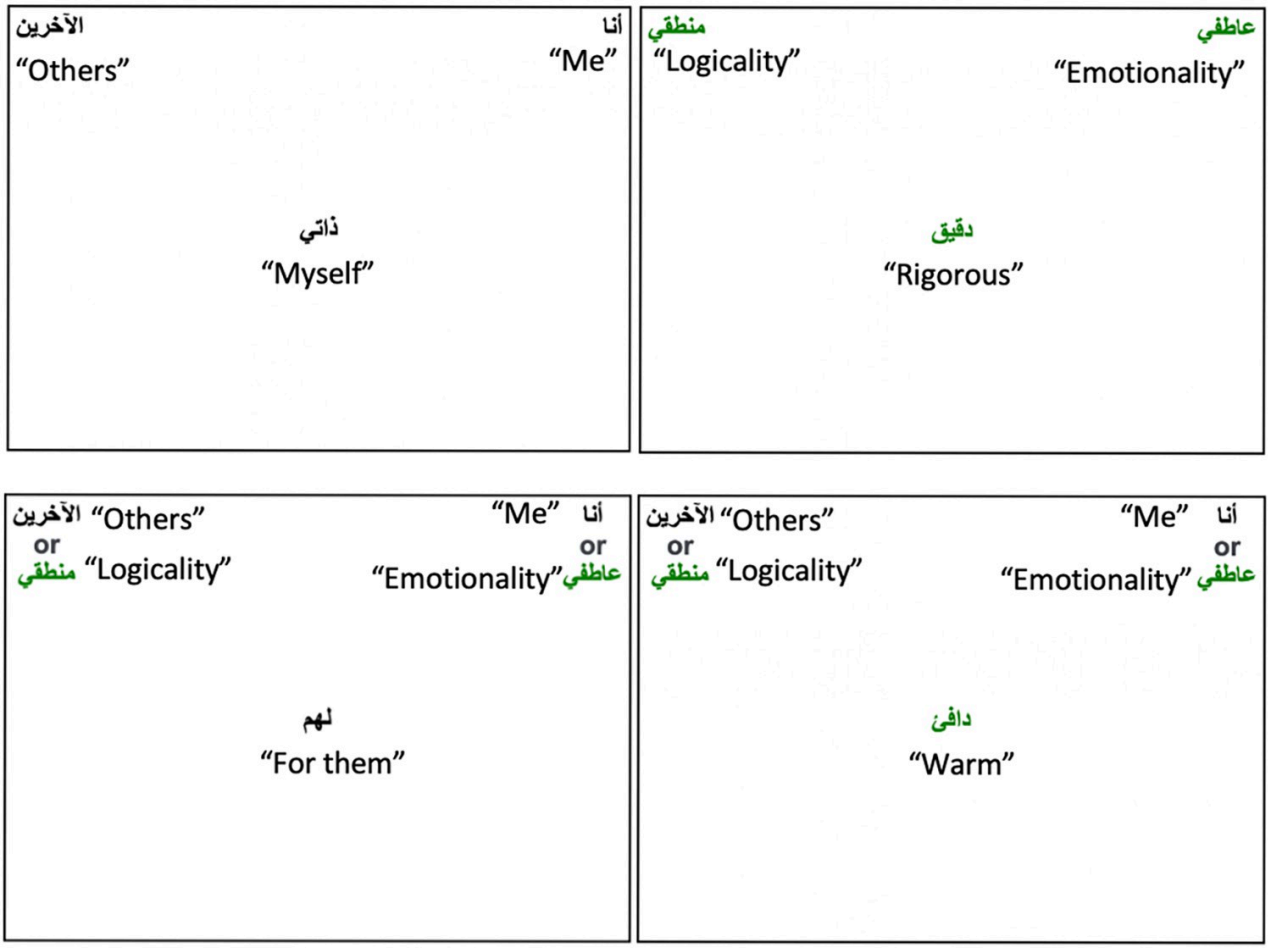
Illustration of emotionality-logicality IAT. *Note*. English translations in quotation marks were not shown to participants.

As shown in Fig 2, two different colours are used for targets and attribute categories. It helps to reduce the task ambiguity when two pairs are shown on a certain task instead of one category on each side, as suggested by Lane et al. [7].

Another stimuli-related concern is the number of stimuli used under each category. The first aspect involves whether an equal number of stimuli should be used under each category. This aspect is under-researched within the field; however, we see no clue in including the unequal number of stimuli under each category. The second aspect is related to the appropriate number of stimuli. Several studies using different numbers of stimuli (e.g., as low as four and as high as 25 stimuli) concluded that stimuli number does not affect the magnitude effect nor the reliability of the scores [3, 13].

Furthermore, the IAT comprises three single-categorisation practice blocks (Blocks 1, 2, and 5) and four double categorisation critical blocks (Blocks 3, 4, 6, and 7). We followed the suggestions in the literature [3, 7, 13] to include 20 trials in Blocks 3 and 6 and 40 trials in Blocks 4 and 7. In Block 5, we included 40 trials to reduce the first combined pair effect on the IAT scores because participants would show greater IAT effects for whichever combined pair is shown first (i.e., order effect) [13, 14]. Also, the IAT was designed through Qualtrics to counterbalance the double categorisation blocks among participants.

Last, as suggested by Lane et al. [7], we included error feedback to the participants whenever the stimulus is wrongly classified (i.e., a red “X” appears in the middle of the screen). The participant is instructed to press the other key to correct his response, and an error penalty is added to his recorded response time.

### The logic behind IAT

In IAT, the task response time is the measurement core, and all conclusions are based on it. As stated earlier, when the IAT was first presented, it was presumed that the participant would perform the classification task more accurately and faster if the associated categories shared the same keys on the keyboard.

For that logic, the response time for each task is recorded and stored. Afterwards, the differences in the responses time to a certain pairing of the target and attribute (e.g., *Me + Emotionality* and *Others + Logicality*) are compared to the reversed set (e.g., *Me + Logicality* and *Others + Emotionality*). This comparison estimates the association strength between the two sets of pairing. To elaborate, if the task response time is faster for the first set, we can conclude that the relative association in the first set is stronger than the other set. Hence, the participants reflect an implicit preference to view themselves as emotional over being logical.

### Scoring and interpreting IAT effects

At early stages, researchers reported the IAT effects as the differences in mean response time between two combined pairings (i.e., Blocks 4 and 7) until Greenwald and colleagues [15] introduced an improved scoring algorithm (called *D*) that overcomes all early IAT scoring methods issues. *D* is the difference in mean response time between the IAT’s two combined tasks (e.g., *Me + Emotionality* and *Me + Logicality*), divided by its associated (i.e., inclusive) standard deviation. They also recommended deleting all trials greater than 10,000 milliseconds (i.e., very slow) and all subjects for which more than 10% of their trials have a response time lesser than 300 milliseconds (i.e., button mashers).

Lane et al. [7] pointed out five advantages of using *D*. First, *D* minimises the correlation between the IAT effects and individuals’ mean response time. Second, it minimises the effect of the IAT blocks’ order. Third, it minimises the effect of IAT experience on selecting new IATs. Fourth, it retains higher internal consistency values. Last, it maximises the correlations between the corresponding implicit and explicit measures. Further, Rudman [2] pointed out that it reduced the unwanted error variance caused by individual differences in performing the task and cognitive skills. Therefore, *D* is used throughout this study during IAT use.

The IAT *D* score is called an IAT effect size. Rudman [2] suggested that the *D* statistic estimates IAT effect size magnitude and the values of.15, .35, and .60 correspond to small, medium, and large effect sizes, respectively. Further, the *D* statistic can be transformed into a more familiar effect size measure, Cohen’s *d*, dividing *D* by the sample’s standard deviation.

### The Big Five personality

Undoubtedly, the five-factor model of personality, known as the Big Five, is the most popular personality model. Although the origin of this model is unclear, it is obvious that this model emerges based on Cattel’s system that depends on the factor analytic approach [16]. The researcher showed that the five-factor model was robust in many studies following the factor analytic approach. In other words, much of what is meant by the term *personality* is explained by the five-factor model.

Nevertheless, the factor names and their interpretations were inconsistent among the researchers. For example, Norman’s [17] five factors were surgency, agreeableness, conscientiousness, emotional stability, and culture. While Costa and McCrae’s [9] most popular five factors were extraversion, agreeableness, conscientiousness, neuroticism, and openness to experience. Frequently, there is a global consensus on factors’ labels: I. Extraversion or surgency; II. Friendliness or agreeableness; III. Conscientiousness; IV. Neuroticism or emotional stability; and V. Openness to experience or intellect.

### Trait emotional intelligence

Trait emotional intelligence (trait EI) is described as a set of emotional perceptions that may be assessed using self-report measures [18]. Because trait EI is primarily concerned with people’s judgements of their emotional capacity, it is often referred to as "trait emotional self-efficacy”. The Trait Emotional Intelligence Questionnaire is used as a vehicle to study the paradigm.

### The Big Five and trait EI

Several studies have been conducted to examine the relationship between the Big Five factors of personality and trait EI [19–29]. This is not surprising given that trait EI is considered as a personality trait and investigating its association with a well-established personality taxonomy such as the Big Five model should be a concerned. In most of these studies, the personality factor of Neuroticism showed the greatest correlation with the global trait EI, among the other Big Five factors of personality. Extraversion and Conscientiousness also showed a relatively stronger correlations with the global trait EI, compared to Agreeableness and Openness.

### The present study

Almost twenty years ago, the concept of implicit personality was introduced to the psychology field by Greenwald and Farnham [8]. However, the concept did not receive much attention in Arab countries like Kuwait. In fact, to our knowledge, there has only been a single implicit personality study in an Arabic country [30]. It investigated aggressive behaviour through the Conditional Reasoning Test, in Egyptian samples. Obviously, the measures used to assess the implicit personality have not yet been introduced to any Kuwaiti sample. This explains why we are interested in conducting the present study in Kuwait.

The overall aim of this study is to introduce a novel approach to assess implicit personality through implicit association test (IAT) in Kuwaiti Arabic for use in general population. Specifically, we were interested in a) adapting the Big Five IAT and b) constructing the trait EI IAT. For the Big Five IAT, we adapted the English Big Five IAT developed by Back et al. [31] into Kuwaiti-Arabic. While for the trait EI IAT, we followed the IAT construction guidelines presented in the introduction, similar to Back’s et al. [31] methodology to construct a personality related IAT. We also aim to examine the relationship between explicit and implicit data in order to look at whether our data supports Nosek and Smyth [32] view. Accordingly, we will test the following hypotheses:

*H1*: *There will be low correlations between trait EI scores obtained by the explicit (TEIQue-SF) and implicit (trait EI IAT) measures*.
*H2*: *There will be low correlations between the Big Five scores obtained by the explicit (NEO-FFI) and implicit (Big Five IAT) measures*.

## Materials and methods

### Pilot study and cultural adaptation design and procedure

The ITC [33] test adaptation guidelines were considered in the adaptation of the English Big Five IAT, and accordingly, an expert committee was formed, with members tasked with forward- and back-translating the measure. First, the stimuli were obtained from the English Big Five IAT version by Back and his colleagues [31]. After that, two committee members translated these stimuli into simple Arabic. The two forms were not identical in terms of identifying the same stimuli in Arabic by the two forward translators. However, this is due to the existence of several synonyms for every word in Arabic. Therefore, these discrepancies were resolved by choosing the most appropriate and cultural-reflective stimuli. Subsequently, a synthesised form was sent to the back-translating team, who, in return, constructed two English forms of the stimuli. The researcher compared the two forms, and the same issue of multiple synonyms, but in English, appeared. The two back-translators followed the same procedures followed by the forward translators in identifying the most appropriate stimuli considering the two versions. All materials were then reviewed by one of the committee members and approved for piloting without any amends.

After piloting the Big Five IAT for the first time, the Agreeableness subtest showed an unacceptable reliability estimate of .42 through the split-half method. We contacted the committee to revise the stimuli, and the same translation procedure was followed again. In the forward translation stage, the committee members decided to use a different synonym of every problematic stimulus within this subtest. After that, the back-translation and the final revision procedures were followed. The committee approved the final version of this subtest and suggested performing a focus group before piloting the subtest again. The participants in the focus group were able correctly to categorise each stimulus under the purposed category without any mistakes. Therefore, we decided to proceed with the revised version of this subtest for the second pilot of the Agreeableness subtest within the Big Five IAT. Accordingly, the final version of the Big Five IAT comprised 5 sub-IATs corresponding to the five factors of: Neuroticism, Extraversion, Openness, Agreeableness, and Conscientiousness.

We also constructed the first trait EI IAT draft following the guidelines by Lane and colleagues [7]. We created a list of stimuli through identifying the appropriate Arabic synonyms that can be classified under each trait EI factor. The list of stimuli was accepted by the expert committee. Thus, four different sub-IATs were constructed corresponding to the four-factor trait EI model proposed by Petrides [10]: Well-being, Self-control, Emotionality, and Sociability. The four trait EI sub-IATs were developed using Qualtrics to pilot it.

After piloting the trait EI IAT, we looked at the reliability estimates obtained through split-half and Cronbach’s alpha methods. The estimates suggested a possible issue with the Emotionality factor of trait EI, as can be seen found in S1 Table. Therefore, we decided to revise the list of stimuli following the same procedures explained above for the Agreeableness sub-IAT.

### Main study design and procedure

We used a convenience sample design and approached participants via an anonymous Qualtrics link (online). These participants differed from those used for the pilot samples, and the final IAT versions were again implemented on the main study sample. Several faculty members within Kuwaiti higher education institutions were contacted individually to help disseminate the Qualtrics link. Participants did not provide any personal self-identifying information, and provided their consent to participate by clicking on “*I consent to participate in this study*”.

The Qualtrics link comprises five sections after consenting to participate in the study. The participants were asked to answer demographic questions in the first section. The second and third sections concerned the explicit and implicit Big Five factor measures, respectively. The fourth and fifth sections concerned the explicit and implicit trait EI measures, respectively.

This study was approved by the University College London-Departmental Ethics Committee (CEHP/2021/586). All research methods were conducted in accordance with relevant guidelines and regulations.

### Participants

The first pilot sample of university students comprises 57 participants completed the trait EI IAT. The second pilot sample of university students comprises 64 participants completed the Big Five IAT. The third pilot sample of university students comprises 34 participants completed the revised Agreeableness sub-IAT. Sample sizes are complied with the suggestions from the measure’s piloting literature [34].

The main study sample included 1458 university students in Kuwait with a mean age of 22.34 years (SD = 7.62 years). The characteristics of our sample can be found in Table 1. We did not identify any missing values in our dataset, and therefore, all participants were included in our study for further analysis.

**Table 1:** The characteristics of our sample (N = 1458).

Variable		n	%
**Nationality**			
	Kuwaiti	1301	9.05%
	Non-Kuwaiti	132	89.23%
	PNS	25	1.71%
**Gender**			
	Female	1110	76.13%
	Male	336	23.05%
	PNS	12	.82%
**Marital Status**			
	Currently married	235	16.12%
	Currently unmarried	1192	81.67%
	PNS	31	2.13%
**Last Degree Obtained**			
	Highschool or below	1124	77.09%
	Post School Diploma	99	6.8%
	Bachelor	232	15.91%
	Masters & PhD	3	.21%
**Household Income**			
	Less than 500 KWD	59	4.05%
	Between 501-1000KWD	203	13.92%
	Between 1001–1500 KWD	205	20.92%
	Between 1501–2000 KWD	247	16.94%
	More than 2000 KWD	310	21.26%
	PNS	334	22.91%
**Major**			
	Art & Humanities	771	52.88%
	Science & Engneering	687	47.12%

*Note*. PNE = Prefer not to say, KWD = Kuwaiti Dinar

### Measures

#### Trait EI IAT

The TEI IAT is a four-IAT subtests reflecting the four factors model proposed by Petrides [10]. The first subtest comprises five separate categorisation tasks, represented by seven blocks. In the other subtests, the first block is eliminated as the task was already done in the first subtest. An illustration of these blocks is presented in Fig 1 using one of the sub-IATs from our study. The IAT is developed following the guidelines by Lane and colleagues [7] as shown earlier in the *Building IAT Blocks section*.

In general, for each block, a mutually exclusive stimulus that belongs to either a left or a right side of the concept will appear in the middle of the participant’s screen. The task asks the participant to classify each stimulus by pressing two pre-specified keys (a left and a right key) on his keyboard. During this classification task, the response time to each stimulus is recorded for further analysis. The list of stimulus and categories used in our study can be found in S1 Appendix.

#### Big Five IAT

The Big Five IAT is a five-IAT subtests reflecting the big five personality dimensions proposed by Costa and McCrae [9]. The English stimulus were obtained from the English Big Five IAT version by Back and his colleagues [31] and the full list of stimulus and categories can be found in S2 Appendix. As we did in the trait EI IAT, only the first subtest included the first block of me + others categorisation task.

#### Kuwaiti Arabic Trait Emotional Intelligence Questionnaire (TEIQue-SF)

The TEIQue-SF is a 30-items inventory providing comprehensive coverage of the sampling domain of trait EI in adults [10]. The items are responded to a 7-point Likert scale. All TEIQue instruments are available, free of charge, for research purposes from www.psychometriclab.com. In this study, we used the Kuwaiti-Arabic adaptation of TEIQue-SF [35], which has shown robust psychometric properties in Kuwaiti samples.

#### Kuwaiti-Arabic NEO-FFI

The NEO-FFI is the short form of the NEO-PI developed by Costa and McCrae [9]. The inventory comprises 60 items providing scores on the Big Five factors: Neuroticism (N), Extraversion (E), Openness (O), Agreeableness (A), and Conscientiousness (C). One limitation is that it does not yield scores at the facet level as the NEO-PI. However, we used it in our study due to circumstances relating to our project (esp., limited time). We used Alansari’s [36] Kuwaiti-Arabic adaptation.

### Data analysis plan

Descriptive statistics and reliability estimates will be calculated using *R 4*.*0*.*5* [37]. The *iatgen* package [38] will be used to perform all analyses. Participants will be considered *button mashers* and dropped from the final analysis if they had too many fast responses (i.e., more than 10% of their responses were below 300 ms).

The reliability estimates will be computed based on creating pairs of reaction times from compatible/incompatible blocks, calculating their differences, and apply them to Cronbach’s alpha analysis [14].

Additionally, we will model the interrelationships between the two implicit constructs (i.e., trait EI and Big Five) through SEM based on the relationships reported in Petrides et al. [25]. The analysis will be carried out through Mplus [39], and the corresponding parameters will be estimated with the robust maximum likelihood estimator (MLR) to deal with deviations from normality.

## Results

### Pilot study results

Three pilot studies were conducted to look at the descriptive statistics and reliability estimates of the scores obtained by the trait EI and the Big Five IATs. The test-related information of these pilot studies can be found in S1 Table. The first pilot study comprised 57 participants who completed the trait EI IAT. The number of participants who completed the test dropped from 57 participants who completed the first subtest to 55 participants who completed the last subtest. Also, the number of button mashers increased as participants approached the end of the overall test. The reliability estimates through the split-half method ranged between .43 and .79. In comparison, the estimates through Cronbach’s alpha ranged between .57 and .70.

The second pilot study comprised 64 participants who completed the Big Five IAT. The results can be found in S1 Table. The number of participants who completed the test dropped from 64 participants who completed the first subtest to 51 participants who completed the last subtest. Also, the number of button mashers increased as participants approached the end of the overall test. The reliability estimates through the split-half method ranged between .42 and .82. In comparison, the estimates through Cronbach’s alpha ranged between .61 and .81.

The third pilot study comprised 34 participants who only completed the revised Agreeableness factor of the Big Five sub-IAT and Emotionality factor of trait EI sub-IAT. The results can be found in S1 Table. Thirty-four participants completed the two revised sub-IAT. The reliability estimate using the split-half and Cronbach’s alpha methods jumped to .83 and .74, respectively, for the Agreeableness sub-IAT. The reliability estimates using the same two methods were .64, and .63, respectively, for the Emotionality sub-IAT. In both cases, the reliability estimates were higher in the revised version.

### Main study results

The IAT-related information for the 9 sub-IATs are shown in Table 2. Descriptive statistics for all variables are shown in Table 3 (N = 1458). All skewness and kurtosis values were within the acceptable ranges (-3.00 to +3.00) and (-10.00 to +10.00), respectively [40].

**Table 2:** The IAT-related information for each sub-IAT (main study).

Subtest		N	Button Mashers ^a^	Error Rate
First Attribute	Second Attribute			
**Big Five IAT **				
Fearlessness** **	Neuroticism	1740	275	.12
Extraversion** **	Introversion	1671	346	.10
Openness** **	Reticence	1622	401	.09
Agreeableness** **	Reluctance	1571	424	.09
Conscientiousness** **	Unscrupulous	1517	466	.09
**Trait EI IAT **				
Sociability** **	Bashfulness	1457	505	.10
Self-control** **	Unrestrainedness	1421	552	.10
Emotionality** **	Logicality	1388	563	.09
Well-being** **	Misery	1357	565	.08

^a^Number of fast participants (Dropped from the analysis).

**Table 3:** Descriptive statistics for the key variables in the main study.

		Overall sample (N = 1458)					Males (N = 336)				Females (N = 1110)			
		Range^a^	M	SD	Skew	Kurt	M	SD	Skew	Kurt	M	SD	Skew	Kurt
**TEIQue-SF**		[1.00–7.00]												
	Global trait EI	2.47–6.80	4.65	0.77	0.26	-0.29	4.75	0.82	0.34	-0.56	4.61	0.76	0.21	-0.25
	Well-being	1.00–7.00	5.26	1.12	-0.38	-0.42	5.26	1.07	-0.17	-0.57	5.26	1.14	-0.44	-0.40
	Self-control	1.00–7.00	4.24	0.98	0.14	0.47	4.45	0.97	0.32	0.28	4.18	0.98	0.09	0.49
	Emotionality	1.75–7.00	4.51	0.87	0.27	-0.02	4.54	0.93	0.28	-0.04	4.49	0.85	0.28	-0.03
	Sociability	1.33–7.00	4.63	1.01	0.08	-0.13	4.76	0.98	0.15	-0.16	4.59	1.02	0.07	-0.14
**NEO-FFI-3**		[12.00–60.00]												
	Neuroticism	14.00–60.00	34.00	5.99	0.45	1.88	33.40	7.32	0.79	1.87	34.20	5.53	0.27	1.43
	Extraversion	16.00–60.00	40.20	5.13	0.09	2.00	41.30	5.39	0.55	1.76	39.90	5.01	-0.11	1.93
	Openness	15.00–60.00	40.30	5.33	-0.04	2.06	41.40	5.87	0.42	1.53	40.00	5.13	-0.32	2.03
	Agreeableness	15.00–60.00	39.90	5.42	0.18	1.72	40.20	5.81	0.85	1.26	39.80	5.32	-0.09	1.77
	Conscientiousness	14.00–60.00	42.50	5.25	-0.44	2.55	42.70	5.16	0.40	1.76	42.50	5.29	-0.67	2.70
**Trait EI IAT**		[-2.00–2.00]												
	Well-being	-.97–1.06	0.22	0.32	-0.26	0.13	0.28	0.33	-0.52	0.96	0.21	0.31	-0.20	-0.06
	Self-control	-.60–1.16	0.23	0.30	-0.09	-0.34	0.25	0.33	0.23	-0.57	0.23	0.29	-0.20	-0.32
	Emotionality	-.89–1.04	0.11	0.30	-0.02	0.18	0.06	0.33	0.05	-0.13	0.11	0.29	-0.03	0.19
	Sociability	-.88–.93	0.05	0.31	-0.11	-0.17	0.15	0.33	-0.30	-0.26	0.02	0.30	-0.11	-0.14
**Big Five IAT**		[-2.00–2.00]												
	Neuroticism	-1.09–1.31	0.27	0.33	-0.21	0.09	0.26	0.36	-0.19	0.18	0.27	0.32	-0.22	0.02
	Extraversion	-1.45–1.38	-0.05	0.43	0.07	-0.28	0.04	0.44	-0.07	-0.21	-0.07	0.42	0.11	-0.29
	Openness	-.83–1.14	0.17	0.34	-0.07	-0.33	0.22	0.35	-0.11	-0.49	0.16	0.33	-0.07	-0.29
	Agreeableness	-.79–1.22	0.32	0.32	-0.26	0.00	0.38	0.35	-0.31	-0.18	0.31	0.31	-0.28	0.04
** **	Conscientiousness	-.99–1.21	0.30	0.33	-0.19	-0.02	0.35	0.33	0.06	-0.47	0.29	0.33	-0.27	0.02

Gender-based reliability estimates for all measures are shown in Table 4. Overall, the implicit measures showed higher reliability estimates compared to their explicit counterparts.

**Table 4:** Gender-based reliability estimates for the key variables in the main study.

		Overall sample (N = 1458)	Males (N = 336)	Females (N = 1110)
		Cronbach’s α	Cronbach’s α	Cronbach’s α
**TEIQue-SF**				
	Global Trait EI	.83	.85	.82
	Well-being	.71	.68	.72
	Self-control	.43	.46	.41
	Emotionality	.44	.53	.41
	Sociability	.52	.49	.53
**NEO-FFI-3**				
	Neuroticism	.77	.76	.75
	Extraversion	.66	.61	.67
	Openness	.31	.16	.33
	Agreeableness	.50	.55	.49
	Conscientiousness	.81	.81	.81
**Trait EI IAT**				
	Well-being	.68	.68	.68
	Self-control	.69	.69	.69
	Emotionality	.64	.64	.64
	Sociability	.72	.72	.72
**Big Five IAT**				
	Neuroticism	.74	.74	.74
	Extraversion	.85	.85	.85
	Openness	.73	.73	.73
	Agreeableness	.71	.71	.71
	Conscientiousness	.71	.71	.71

### The relationship between explicit-implicit constructs

The zero-order correlations between the explicit-implicit constructs are shown in Table 5. For the trait EI factors, they ranged from -.01 to .11 (overall sample), -.02 to .16 (males), and .01 to .10 (females). Similarly, low values were observed for the Big Five factors. These correlations ranged from—.03 to .10 (overall sample), -.06 to .17 (males), and -.02 to .08 (females). Thus, the results showed that our two hypotheses were borne out by our data.

**Table 5:** Gender-based correlations between implicit and explicit constructs.

		Overall sample (N = 1458)	Males (N = 336)	Females (N = 1110)
**Trait EI**				
	Well-being	.11**	.16	.09*
	Self-control	.10**	.09	.10**
	Emotionality	- .01	- .08	.01
	Sociability	.03	- .02	.02
**Big Five**				
	Neuroticism	.05	.17**	.02
	Extraversion	.10***	.17**	.07*
	Openness	- .07*	- .09	- .08*
	Agreeableness	- .03	- .06	- .02
	Conscientiousness	.08*	.09	.08*

Note.

* p < .05

** p < .01

*** p < .001

## Discussion

### Objectives and hypotheses

In this study we aimed to introduce the implicit personality concept to the Kuwaiti psychology field accompanied by adapting and constructing their measurement method. In the introduction, we distinguished between the concepts of explicit and implicit personality through their measurement methods. For instance, explicit personality is assessed through self-report measures by asking the participants to directly choosing a response from a given scale. While implicit personality is assessed indirectly using certain type of tests like the IAT.

Accordingly, we adapted one IAT to assess the Big Five factors indirectly (i.e., implicitly) in Kuwaiti sample. In more details, we adapted the five sub-IATs corresponding to the five-factor model of personality proposed by Costa and McCrae [9]. The stimuli we used in this test were obtained from Back et al. [31]. Also, we considered their methodology along with Lane’s et al. [7] guidelines to construct the first trait EI IAT in the literature as shown in the present study. Through these adapted and newly designed measures, we successfully measured the implicit constructs to test the hypotheses we advanced earlier.

First, we assessed the psychometric properties of our IAT measures. Our reliability analysis yielded satisfactory estimates; however, we believe that the very meaning of internal consistency is questionable within the IAT context. This is because these estimates within the IAT context refer to whether responses time between trials are consistent or not, rather than whether the actual D-scores are consistent [38].

Our results showed that, in general, reliability estimates for implicit measures were higher than for explicit measures. This is at odds with other research showing that explicit measures of personality tend to have higher reliabilities than implicit measures [12, 41, 42]. However, the cross-cultural dimension of our study is a potential confounding factor and further work will be necessary on this point.

### Main findings of our study

We examined the relationships between the explicit and implicit measures of trait EI and the Big Five, separately, through zero-order correlations. The results supported our earlier hypotheses that we will find weak correlations between the scores obtained by explicit and implicit measures for both the Big Five factors and the four trait EI factors. These results are consistent with Lane et al. [7] who reported similarly low correlations between explicit and implicit personality measurements across numerous constructs. It appears that these two methodologies tap into two distinct aspects of personality. Thus, they can be seen as complementary methodologies, that offers full understating of both personality aspects (explicit and implicit), rather than two alternative methodologies for the assessment of personality.

Given the fact that this type of measure is introduced for the first time to Kuwaiti sample, we expected that the error rate will be relatively high in the first sub-IAT of each construct as shown by our results. However, the error rate decreased continuously as the participants approached the end of the IAT. This suggests that experiencing more IATs can help the participant to perform the categorisation tasks more accurately.

### The implications of these results

The implications of the current study are manifold. First, concerning the field of personality, this study contributes to the broad personality literature in two unique ways. The first contribution is our attempt to construct the first trait EI IAT based on Petrides’s [10] four-factor of trait EI model. This is highly novel in the broad implicit personality literature and also in the trait EI literature. The reliability results obtained from our trait EI IAT were promising, however, the validity of the results is still questionable and further investigations shall be made by future researchers. The second contribution is our finding of lack of correlations between the scores obtained from the implicit measures administered in our study and their explicit counterparts. Lane et al. [7] found similarly low correlations between the two measurement types in their review of numerous studies. Clearly, our findings support the view that implicit personality represents a unique aspect of one’s personality. Researchers should not view implicit measures as an alternative way of measuring the same construct, but as two distinct personality aspects (i.e., traits).

Second, concerning policymakers, we believe that we have introduced a novel implicit personality measurement method, which can help us to identify personality traits that people are unwilling to report through self-report measures (i.e., explicit measures). This will benefit policymakers in Kuwait as well as the international context because several organisations rely on psychological measures and other job-related qualifications to hire certain staff members (e.g., leaders). In fact, this is not surprising as certain personality traits (e.g., trait EI) showed significant predictive effects on job performance [43]. Accordingly, some participants may provide desirable responses to obtain higher trait EI scores [44] in order to secure a position in a certain job. Therefore, there is a need for an indirect measurement tool that can offer another view of one’s personality traits, which was offered in the current study.

### The limitations of this study

While our study findings are robust, it is important to acknowledge the limitations of the study in order to provide a balanced and accurate interpretation of the results. In this study, we relied on a non-probability sampling method (i.e., convenience sampling method). Although it was the most appropriate for our study design because we wanted to recruit as large as possible a sample size with restricted time frame and funds [45]. Yet, these methods have a high degree of bias, which threatens the validity of the results obtained in our studies. In addition, it limits us from generalising our findings to the general population.

Another significant limitation in our study is the length of the included measures. Ben-Nun [11] suggested that long measures can lead to a phenomenon called respondent fatigue. This phenomenon occurs when participants become exhausted of the measure’s task, and therefore, affects the quality of the data provided by them. Perhaps, one way to mitigate it is by decreasing the number of tasks without affecting the quality of the measure. Accordingly, we only included the first categorisation task in the first sub-IAT of both trait EI and the Big Five and eliminated this task from the following sub-IATs.

We presented the number of participants who completed each sub-IATs along with the number of button mashers from pilot and main studies. The results from all studies showed that the number of participants was decreasing as they approach the end of the IAT. Also, the number of button mashers was increasing as the participants approached the end of the IAT. Thus, suggesting that the respondent fatigue has occurred in these participants, even though we decreased the number of tasks to mitigate this phenomenon. As a further step, we eliminated these participants from our data analyses in order to avoid any threats to the validity of our results.

Besides the aforementioned attempts to control the respondent fatigue, we also ensured that the instructions given to the participants were clear and concise. This is also to ensure that participants do not spend much of the study time in reading the instructions.

### Future lines of research

In this study, we provided comprehensive tools to assess implicit personality. The measures presented here were adapted and validated following the most comprehensive and updated cross-cultural adaptation guidelines. This, in fact, is also a big contribution to Kuwaiti psychology, as it promotes the research of these novel constructs across different settings in the country.

Two more important directions can also be pinpointed from the results of the current study. As we discussed earlier, one is the need to rethink about the concept of reliability (esp., internal consistency) of implicit data. That said, future researchers should revisit the meaning of the reliability in this context. Also, they should consider coming up with a better methodology to assess and interpret the internal consistency of the scores. Another direction is the study of the meaning of the implicit aspects of the Big Five and trait EI. Our results clearly indicated no correlation between explicit and implicit measures, which raises the question of what implicit personality reflects. We believe that they refer to the unconscious and automatic aspects of a person’s personality, which are deeply ingrained in an individual and unconsciously influence their behaviour and thought patterns. Frankly, we believe that these two directions can be thought of as research projects in their own night.

## Conclusions

Taking altogether, we believe that this study makes a contribution by introducing implicit association test as a vehicle to assess implicit personality constructs in Kuwait. These implicit personality assessment methodologies are highly novel and original in Kuwaiti Psychology field. Not only in Kuwait, but the findings presented in the present study also contributes to the growing implicit personality literature, as we presented results from a relatively large sample. These results supported the idea that the implicit aspect of personality should be thought of as a distinct aspect of personality. Thus, researchers within the field should view it as a different aspect of human personality rather than only viewing it as another method to measure personality.

## Supporting information

S1 Table The iat-related information for each sub-IAT (pilot studies). (DOCX)

S1 Appendix List of stimuli used for the trait EI IAT. (DOCX)

S2 Appendix List of stimuli used for the Big Five IAT. (DOCX)
