# Supplementary material for: The relationship between explicit and implicit personality: Evidence from the Big Five and trait emotional intelligence
Source: PLoS One. 2023 Oct 9;18(10):e0287013. doi: 10.1371/journal.pone.0287013 (PMC10561833; doi:10.1371/journal.pone.0287013)
Supplement: S1 Table — (DOCX) [file pone.0287013.s001.docx]

**Appendix A.** The IAT-Related Information for Each Sub-IAT (Pilot Studies)

| **Subtest** | | **N** | **Button Mashers ^a^** | **Error Rate** | **Split-half** | **α** |
| --- | --- | --- | --- | --- | --- | --- |
| **First Attribute** | **Second Attribute** |  |  |  |  |  |
| **Pilot Sample 1: Trait EI IAT** | |  |  |  |  |  |
| Sociability | Bashfulness | 57 | 0 | .11 | .79 | .70 |
| Self-control | Unrestrainedness | 55 | 2 | .08 | .61 | .66 |
| Emotionality | Logicality | 55 | 5 | .09 | .43 | .57 |
| Well-being | Misery | 55 | 4 | .08 | .53 | .70 |
| **Pilot Sample 2: Big Five IAT** | |  |  |  |  |  |
| Fearlessness | Neuroticism | 64 | 4 | .09 | .82 | .79 |
| Extraversion | Introversion | 60 | 5 | .09 | .71 | .78 |
| Openness | Reticence | 54 | 4 | .07 | .71 | .61 |
| Agreeableness | Reluctance | 52 | 7 | .08 | .42 | .81 |
| Conscientiousness | Unscrupulous | 51 | 7 | .08 | .63 | .66 |
| **Pilot Sample 3: Revised IATs^b^** | |  |  |  |  |  |
| Agreeableness | Reluctance | 34 | 0 | .11 | .83 | .74 |
| Emotionality | Logicality | 30 | 2 | .11 | .64 | .63 |

**^a^Number of fast participants (Dropped from the analysis).**
