# Supplementary material for: The relationship between explicit and implicit personality: Evidence from the Big Five and trait emotional intelligence
Source: PLoS One. 2023 Oct 9;18(10):e0287013. doi: 10.1371/journal.pone.0287013 (PMC10561833; doi:10.1371/journal.pone.0287013)
Supplement: S1 Appendix — (DOCX) [file pone.0287013.s002.docx]

**Appendix B.** List of stimuli used for the Trait EI IAT

Me: I, me, my, mine, self

Others: Others, they, them, their, it

Emotionality: sociable, talkative, active, impulsive, outgoing

Logicality: rigorous, reticent, passive, deliberate, reserved

Self-control: stable, calm, cautious, flexible, motivated

Unrestrainedness: irritable, stressed, impetuous, inflexible, demanding

Sociability: leaders, influencers, confident, social

Bashfulness: followers, overwhelmed, uncertain, shy

Well-being: positive, optimistic, cheerful, happy

Misery: negative, pessimistic, gloomy, sad
