# Supplementary material for: The relationship between explicit and implicit personality: Evidence from the Big Five and trait emotional intelligence
Source: PLoS One. 2023 Oct 9;18(10):e0287013. doi: 10.1371/journal.pone.0287013 (PMC10561833; doi:10.1371/journal.pone.0287013)
Supplement: S2 Appendix — (DOCX) [file pone.0287013.s003.docx]

**Appendix C.** List of stimuli used for the Big Five IAT

Me: I, me, my, mine, self

Others: Others, they, them, their, it

Neuroticism: anxious, nervous, fearful, uncertain, afraid

Fearlessness: calm, relaxed, restful, at ease, balanced

Extraversion: sociable, talkative, active, impulsive, outgoing

Introversion: shy, reticent, passive, deliberate, reserved

Openness: imaginative, civilized, well-educated, interested, gifted

Reticence: unimaginative, primitive, uneducated, indifferent, limited

Agreeableness: trusting, well-meaning, friendly, helpful, goodnatured

Reluctance: obstinate, quarrelsome, hostile, hard-hearted, resentful

Conscientiousness: meticulous, reliable, neat, fussy, thorough

Unscrupulous: careless, unreliable, chaotic, frivolous, erratic
